# Hereditary Breast Cancer: Comprehensive Risk Assessment and Prevention Strategies

**DOI:** 10.3390/genes16010082

**Published:** 2025-01-13

**Authors:** Eliza Del Fiol Manna, Davide Serrano, Laura Cazzaniga, Sara Mannucci, Cristina Zanzottera, Francesca Fava, Gaetano Aurilio, Aliana Guerrieri-Gonzaga, Matilde Risti, Mariarosaria Calvello, Irene Feroce, Monica Marabelli, Cecilia Altemura, Lucio Bertario, Bernardo Bonanni, Matteo Lazzeroni

**Affiliations:** 1 Division of Oncogenetics, Unimed Sorocaba, Sorocaba 18040-580, Brazil; elizadelfiol@gmail.com; 2 Division of Cancer Prevention and Genetics, IEO European Institute of Oncology IRCCS, 20141 Milan, Italy; davide.serrano@ieo.it (D.S.); laura.cazzaniga@ieo.it (L.C.); sara.mannucci@ieo.it (S.M.); cristina.zanzottera@ieo.it (C.Z.); francesca.fava@ieo.it (F.F.); gaetano.aurilio@ieo.it (G.A.); aliana.guerrierigonzaga@ieo.it (A.G.-G.); matilde.risti@ieo.it (M.R.); mariarosaria.calvello@ieo.it (M.C.); irene.feroce@ieo.it (I.F.); monica.marabelli@ieo.it (M.M.); cecilia.altemura@ieo.it (C.A.); lucio.bertario@gmail.com (L.B.); bernardo.bonanni@ieo.it (B.B.); 3 Department of Health Sciences, Medical Genetics, University of Milan, 20122 Milan, Italy; 4 Oncology Competence Center, Gruppo Ospedaliero Moncucco, 6900 Lugano, Switzerland

**Keywords:** hereditary breast cancer, risk assessment, prevention

## Abstract

Women carrying pathogenic/likely pathogenic (P/LP) variants in moderate- or high-penetrance genes have an increased risk of developing breast cancer. However, most P/LP variants associated with breast cancer risk show incomplete penetrance. Age, gender, family history, polygenic risk, lifestyle, reproductive, hormonal, and environmental factors can affect the expressivity and penetrance of the disease. However, there are gaps in translating how individual genomic variation affects phenotypic presentation. The expansion of criteria for genetic testing and the increasing utilization of comprehensive genetic panels may enhance the identification of individuals carrying P/LP variants linked to hereditary breast cancer. Individualized risk assessment could facilitate the implementation of personalized risk-reduction strategies for these individuals. Preventive interventions encompass lifestyle modifications, chemoprevention, enhanced surveillance through breast imaging, and risk-reducing surgeries. This review addresses the current literature’s inconsistencies and limitations, particularly regarding risk factors and the intensity of preventive strategies for women with P/LP variants in moderate- and high-penetrance genes. In addition, it synthesizes the latest evidence on risk assessment and primary and secondary prevention in women at high risk of breast cancer.

## 1. Introduction

Female breast cancer is the most diagnosed cancer and the leading cause of cancer death in women worldwide [1]. One in eight women (12.9%) will develop breast cancer in their lifetime [2]. Early detection of breast cancer in asymptomatic women through screening is an essential strategy for reducing the burden of breast cancer [3].

Breast cancer is a multifactorial disease, and its occurrence depends on genetic and nongenetic factors [4]. Around 70–75% of breast cancer cases are sporadic [4]. Lifestyle, mammographic breast density, proliferative breast disease, and hormonal, reproductive, and environmental factors may explain the occurrence of sporadic breast cancer [5]. Familial breast cancer accounts for 20–25% of the cases and shows a higher incidence of breast cancer in a family than observed in the general population and a variable age of onset in the absence of other typical features of hereditary cancer syndromes [6]. Familial breast cancer may occur by a chance clustering of sporadic cases, common genetic background, single-nucleotide-polymorphism (SNP) profile, shared environment, lifestyle choices, or a combination of these factors [6]. Hereditary breast cancers show a dominant inheritance pattern and derive from pathogenic/likely pathogenic (P/LP) variants in breast cancer susceptibility genes, comprising only 5–10% of breast cancer cases [6].

Women carrying P/LP variants in moderate- or high-penetrance genes, such as *BRCA1*, *BRCA2*, *PALB2*, *TP53*, *CDH1*, *ATM*, *CHEK2*, *NF1*, *PTEN*, *BARD1*, *RAD51C*, *RAD51D*, and *STK11* genes, have an increased risk of developing hereditary breast cancer [7]. The penetrance of a P/LP variant refers to the proportion of individuals carrying the variant will actually develop breast cancer during their lifetime [8]. The risk of breast cancer associated with P/LP variants differs by gene, by specific gene locus, and by penetrance (low < 20%, moderate 20–50%, and high penetrance > 50%) [9]. Moderate-risk breast cancer alleles confer an increased risk for breast cancer of two to fourfold compared to the risk of the general population; high-risk alleles confer a risk higher than fourfold the population risk [10].

In population-based studies, *BRCA1* and *BRCA2* P/LP variants were associated with a significantly increased risk of breast cancer, with an odds ratio (OR) ranging from 7.62 to 10.57 and 5.23 to 5.85, respectively [7,11]. The risk associated with breast cancer for P/LP variants in PALB2 varied between moderate and high (OR 3.83–5.02), whereas those in *CHEK2* and *ATM* were associated with moderate risk (OR 2.54–2.47 and OR 1.82–2.10, respectively) [7,11].

Most P/LP variants associated with an increased risk for breast cancer show incomplete penetrance [12]. Approximately half of the individuals who carry P/LP variants in *BRCA1* and *BRCA2* genes do not have a suggestive family history [13]. In addition, according to recent data, *BRCA1/BRCA2* de novo mutation rate estimates are low, around 0.3% [14]. The same P/LP variant can cause different phenotypes, even among related subjects of the same family [15]. Therefore, individuals carrying moderate- or high-risk P/LP variants present different risks of breast cancer depending on the germline variant type, family history, lifestyle, polygenic risk scores (PRS), and other factors [7]. However, there are gaps in translating how the individual genomic variation affects phenotypic presentation and how genetic variants exert their functional impact to cause disease [15].

Hereditary breast cancer is often underdiagnosed because current testing guidelines miss a considerable number of patients with breast cancer carrying clinically actionable P/LP variants. A cohort study evaluated 29 genes with actionable NCCN management guidelines (v.2 2024) in 195,615 consecutive patients (aged 18–100 years) who underwent hereditary cancer testing between June 2020 and August 2023. The results showed that around 40% of patients with P/LP variants did not meet the current NCCN testing criteria [16].

Adopting broader criteria for genetic panel testing may raise the possibility of identifying P/LP variants associated with hereditary cancer in breast cancer patients and their family members through cascade screening [17]. Furthermore, the increasing use of expanded genetic panels has enabled the identification of clinically relevant P/LP variants, many of which were not associated with personal or family history of breast cancer [17].

Individualized risk assessment helps women at increased risk for breast cancer to adopt personalized risk-reduction strategies to reduce breast cancer occurrence and mortality [18]. Risk-reduction interventions for hereditary breast cancer include lifestyle modification, chemoprevention, intensified surveillance with breast imaging, and risk-reducing surgeries [19]. This review summarizes the most recent evidence regarding risk assessment and primary and secondary prevention in women carrying P/LP variants in moderate- and high-penetrance genes.

## 2. Risk Assessment

Different factors can act as genetic modifiers, affecting gene penetrance and expressivity. These factors include age, gender, family history, PRS, lifestyle choices (e.g., alcohol consumption and physical activity), breast density, and hormonal (e.g., hormone replacement therapy, HRT), reproductive (e.g., breastfeeding), and environmental influences (e.g., exposure to ionizing radiation) [20]. Risk assessment for hereditary breast cancer should be dynamic, incorporating factors beyond genetics to improve predictive accuracy. Several consensus statements and guidelines have been developed to recommend personalized risk assessment and preventive strategies for P/LP variant carriers [21,22].

### 2.1. Age

The penetrance of a gene may vary according to the patient’s age. Kuchenbaecker et al. showed that the cumulative breast cancer risk by the age of 80 was 72% for *BRCA1* and 69% for *BRCA2* P/LP variant carriers; breast cancer incidence increased rapidly until the ages of 35 to 40 for *BRCA1* and until the ages of 40 to 50 for *BRCA2* carriers, and then the risk tends to stabilize until age 80 [23].

Fan et al. studied the penetrance and clinical outcomes of seven breast cancer susceptibility genes (ATM, BRCA1, BRCA2, CHEK2, PALB2, PTEN, and TP53) in 13,458 participants unselected for personal or family history of breast cancer. A P/LP variant in one of the seven genes was identified in 242 female participants [12]. The results showed an association between BRCA1 and TP53 and early-onset breast cancer diagnosed before age 50 years. Conversely, BRCA2, ATM, PALB2, and CHEK2 were more commonly associated with later-onset breast cancer, for which the penetrance estimates ranged from 19% to 31% by age 60 years [12]. However, data on risks for older P/LP variant carriers are often limited [24].

### 2.2. Gender

Penetrance also differs by gender. P/LP variants in *BRCA1*, *BRCA2*, *PALB2*, *CHEK2*, and *ATM* have been associated with male breast cancer [25]. Meijers-Heijboer et al. found that the *CHEK2-1100delC* variant resulted in an approximately twofold increase in breast cancer risk in women and a tenfold increase in risk in men [26]. In a retrospective analysis of 715 cases of male breast cancer, *BRCA2* and *CHEK2* were the most frequently involved genes in hereditary male breast cancer [27].

### 2.3. Family History

Studies have shown that family history significantly affects the penetrance of breast cancer in P/LP variant carriers and noncarriers [28,29]. A strong association between the risk of breast cancer and family history was found for *ATM*, *BRCA1*, *BRCA2*, *CHEK2*, and *PALB2* P/LP variant carriers [24,30].

Jackson et al. reported a significant difference in breast cancer risk between women with and without a family history for *BRCA1* (HR 10.29 vs. 7.24) and *BRCA2* P/LP variant carriers (HR 7.82 vs. 4.66, respectively). Moreover, penetrance to age 60 for *BRCA1* and *BRCA2* P/LP variant carriers was higher in those women with a family history (44.7%, 95% CI 32.2–59.3, and 24.1%, 95% CI 17.5–32.6, respectively) versus those without one (22.8%, 95% CI 15.9–32.0, and 17.9%, 95% CI 13.8–23.0, respectively) [31].

Antoniou et al. reported an absolute risk of breast cancer for women carrying P/LP variants in *PALB2* by the age of 70 of 33% (95% CI 25–44) for those with no affected relatives and 58% (95% CI 50–66) for those with two first-degree relatives with breast cancer diagnosed by the age of 50 [32]. In *CHEK2* carriers with no affected relatives, the risk of breast cancer was approximately 20%, increasing to 44% when both first- and second-degree relatives were affected [33].

A limited family structure, defined as fewer than two first- or second-degree female relatives surviving beyond 45 in either lineage, can also affect the accuracy of mutation probability models and risk assessment [34]. Breast cancer risk estimates for individuals with a limited family structure may be higher than those with a large family. For these reasons, genetic counseling and risk assessment should always consider family size, age, and the number of female relatives.

### 2.4. The Effect of Ascertainment Bias

Ascertainment bias is a systematic error in disease risk estimation caused by the methods used to collect data [35]. It occurs because study participants are often selected non-randomly, with individuals who have a strong family history of breast or ovarian cancer being more likely to undergo genetic testing and participate in studies [36]. As a result, risk estimates based on data from selected BRCA families may not reflect the average risk for all P/LP variant carriers [24]. Therefore, breast cancer risk estimates for BRCA P/LP variant carriers with a family history are higher than those derived from the general population [9,35,37].

For example, studies focusing on breast cancer patients and their families estimated the penetrance of BRCA1 and BRCA2 mutations by age 70 to be 65–85% and 70–84%, respectively [21,38,39]. In contrast, the CARRIERS (Cancer Risk Estimates Related to Susceptibility) study, which included individuals both with and without a strong family history of breast or ovarian cancer, reported breast cancer risk estimates of less than 50% by age 70 for carriers of P/LP variants in BRCA1 and BRCA2 [11].

Li–Fraumeni syndrome (LFS) is a rare hereditary cancer predisposition syndrome characterized by germline P/LP variants in the TP53 gene, which encodes the p53 tumor suppressor protein [40]. This syndrome is associated with a high lifetime risk of developing various types of cancers, often at a young age, including breast cancer [40].

The expressivity and penetrance of Li–Fraumeni syndrome (LFS) may vary according to the genetic testing criteria adopted to establish the diagnosis [41]. Kratz et al. showed significant differences in tumor spectra between *TP53* P/LP variant carriers who met the LFS genetic testing criteria and those who did not [41]. In particular, *TP53* P/LP variant carriers who met the genetic testing criteria had more early adrenal, brain, and bone tumors, whereas those who did not meet had more liposarcoma, glioblastoma, breast, ovarian, pancreatic, and skin cancers [41]. Moreover, tumors diagnosed in individuals who did not meet the LFS genetic testing criteria occurred at more advanced ages, suggesting a less penetrant phenotype [41]. Thus, risk assessment and genetic counseling should consider the occurrence of *TP53* P/LP variants in women with no criteria suggestive of LFS [42].

### 2.5. Other Genetic Factors

The evidence on breast cancer risk for most genes relates to protein-truncating variants leading to a loss of gene function [7,24]. Moreover, variant-specific differences in risk exist even among protein-truncating variants [43]. For the *BRCA1*, *BRCA2*, *PALB2*, *CHEK2*, and *ATM* genes, most protein-truncating variants have been associated with a high risk of breast cancer [24,35,44]. For *PALB2*, most studies have assessed only protein-truncating variants, and little evidence is available for missense variants [7,11,45].

Most missense variants in the *BRCA1*, *BRCA2*, and *ATM* genes do not confer an increased risk of breast and ovarian cancer, but ones in specific domains do [46,47]. For instance, the missense pathogenic variant c.7271T>G in *ATM* may considerably increase the risk of breast cancer in a similar proportion as P/LP variants in *BRCA2* [48]. Most missense variants in *BRCA2* remain classified as variants of uncertain significance (VUSs) because of scarce phenotype and genotype information [49]. Functional assays have shown that *BRCA2* missense variants identified as functionally aberrant and classified as pathogenic under the American College of Medical Genetics and Genomics/Association for Molecular Pathology (ACMG/AMP) guidelines were associated with a slightly lower risk of breast cancer than protein-truncating variants in the *BRCA2* DNA-binding domain (OR 5.15; 95% CI 3.43–7.83 versus OR 8.56; 95% CI 6.03–12.36) [49].

The gene location of P/LP variants is also associated with different risks of breast cancer. Kuchenbaecker et al. reported an increased risk of breast cancer for P/LP variants located outside versus within the regions bounded by positions c.2282-c.4071 in *BRCA1* and c.2831-c.6401 in *BRCA2* (HR 1.93; 95% CI 1.36–2.74; *p* < 0.001) [23]. Another study showed an association between a nonsense variant at the carboxyl terminus of *BRCA2* (p.Lys3326Ter) with a relative risk of breast cancer of 1.4, substantially lower than the risk conferred by more proximal truncating variants (RR = 11.7) [50]. Pal et al. recently identified a clinically significant subset of sixteen *BRCA1* and *BRCA2* variants associated with a moderate two- to four-fold increase in breast cancer risk. These variants function as susceptibility alleles with reduced penetrance, in contrast to complete loss-of-function mutations, such as protein-truncating variants [51]. Consequently, genotype–phenotype correlation may impact the risk management discussion [52].

The role of germline copy number variants (CNVs) as breast cancer risk modifiers in women carrying *BRCA1* and *BRCA2* P/LP variants remains relatively unknown [53]. Germline CNVs overlapping *BRCA1* and *BRCA2* gene loci associated with the pathogenesis of breast cancer account for less than 5% of known P/LP variants in these genes [53]. In a genome-wide association study of CNVs in 2500 *BRCA1* carriers, 52 gene loci were associated with an increased risk of breast cancer [54].

Epigenetics refers to dynamic and heritable modifications that occur in the genome independently of changes in the DNA sequence [55]. Epigenetic alterations in breast cancer development may affect genes involved in proliferation, cell motility, invasion, and apoptosis [55]. The DNA methylation profile may reflect both acquired and inherited risk markers from diverse personal exposures [56]. Thus, epigenetic markers of breast cancer could be integrated into individual genetic profiles and current risk prediction models to assess their interactions and enable target risk reduction strategies.

It is unclear whether individuals with overlapping P/LP variants (double heterozygosity) would show a more severe phenotype [57]. Carrying *BRCA1* and *BRCA2* P/LP variants in a single patient is uncommon, except in specific subpopulations with founder P/LP variants [58]. The percentage of double heterozygosity varies between 0.2% and 0.8% in different ethnic groups, reaching 1.8% in Ashkenazi Jewish people [58,59].

However, the clinical outcome of women carrying *BRCA1* and *BRCA2* P/LP variants seems to be similar to that of patients with a single *BRCA1* P/LP variant, and the probability of developing breast and ovarian cancers is comparable to those with single mutations [57,60]. In *CHEK2-1100delC* carriers, the risk of breast cancer did not change in women also carrying P/LP variants in *BRCA1*, possibly because *CHEK2* and *BRCA1* may function in the same biological pathway [26,33]. The management of individuals carrying double heterozygosis should consider the P/LP variant with the highest penetrance and combine preventive measures for different cancers of both hereditary syndromes [57].

Some germline P/LP variants in cancer predisposition genes have shown a dose effect, resulting in a more severe phenotype in homozygous than heterozygous cases [61]. For *CHEK2* P/LP variants, the homozygous state confers a higher risk of breast cancer than the heterozygous state [62,63,64,65]. Cancer-predisposing P/LP variants in tumor suppressor genes display different phenotypes in heterozygous and homozygous states [61]. For instance, heterozygosis in *PALB2*, *BRIP1*, and *ATM* increases the lifetime risk of breast cancer, while homozygosis causes multisystemic genetic syndromes, such as Fanconi’s anemia (*PALB2* and *BRIP1*) and ataxia-telangiectasia (*ATM*) [66,67,68].

Genome-wide association studies (GWASs) showed an association between the combination of the effect of various lower-penetrance gene variants and an increased risk of breast cancer [69,70]. The combined risk of various SNPs associated with breast cancer risk may explain over 30% of the breast cancer heritability [69,71,72]. Studies conducted by the Breast Cancer Association Consortium, which included European populations, were able to develop the PRS_313_, based on 313 breast cancer-associated variants that allowed them to stratify women according to their risk of breast cancer [73].

PRSs have also been incorporated in risk prediction of hereditary cancer to better estimate the risk of breast cancer and enable a more personalized risk assessment [73,74].

A retrospective case–control study used data on 150,962 women tested with a multigene hereditary cancer panel to evaluate an 86-SNV PRS in *BRCA1*, *BRCA2*, *CHEK2*, *ATM*, and *PALB2* P/LP variant carriers [75]. The results showed an association between the 86-SNV PRS and breast cancer risk for each of the carrier populations: *BRCA1* (OR 1.20; 95% CI 1.10–1.32), *BRCA2* (OR 1.23; 95% CI 1.12–1.34), *ATM* (OR 1.37; 95% CI 1.21–1.55), and *PALB2* (OR 1.34; 95% CI 1.16–1.55). However, the strongest association was observed for noncarriers (OR 1.47; 95% 1.45–1.49) and *CHEK2* P/LP variant carriers (OR 1.49; 95% CI 1.36–1.64) [75].

## 3. Genetic Risk Models That Include Individuals with Hereditary Cancer

Breast cancer risk assessment tools are numerical models designed to estimate the probability or risk of developing breast cancer either over a fixed time horizon (e.g., five or ten years) or from the time of evaluation to older age [3,76,77,78]. Previous risk-assessment tools considered information on reproductive factors (e.g., age at menarche/menopause, age at first live birth), breast biopsies, and family history, whereas later tools incorporated additional lifestyle information, such as menopausal hormone replacement therapy (HRT), alcohol consumption, smoking, and anthropometric data [3].

Risk prediction models may also help to assess breast cancer risk in individuals with inherited P/LP variants. The increasing focus on genetic changes as factors in breast cancer risk highlights the significance of genetic-based risk models [79]. Recent tools have incorporated PRS and P/LP variants in *BRCA1*, *BRCA2*, and other genes [80,81]. The Tyrer–Cuzick, BOADICEA, and ASK2ME models included P/LP variant carriers to calculate breast cancer risk [80,81,82]. The BRCA-Crisk tool helps us to estimate the risk of contralateral breast cancer (CBC) in *BRCA P*/LP variant carriers [83]. Table 1 presents the general characteristics of the genetic risk models available for hereditary breast cancer.

The Tyrer–Cuzick or International Breast Intervention Study (IBIS) model includes extensive family history, comprehensive data on risk factors, and information on *BRCA1/BRCA2* P/LP variants [84].

The BOADICEA (Breast and Ovarian Analysis of Disease Incidence and Carrier Estimation Algorithm) or CanRisk is a genetic risk model based on breast genetic susceptibility due to the effects of the *BRCA1* and *BRCA2* P/LP variants and the assumption that residual clustering within families arises from the multiplicative effects of a polygenic component [85]. Unlike other models, BOADICEA does not limit family history to certain relatives or degrees [86].

ASK2ME.org (All Syndromes Known to Man Evaluator) is a clinical decision support tool that provides absolute cancer risk predictions for various hereditary susceptibility genes [82,87]. The predictions are specific to the patient’s gene carrier status, age, and history of relevant prophylactic surgery [87].

The BRCA-Crisk is a prediction model validated to discriminate *BRCA1* and *BRCA2* carriers with a high risk of CBC from those with low risk and help patients to decide between unilateral versus bilateral mastectomy [83]. The nomogram enables the prediction of the possible 5- or 10-year cumulative risk of CBC for *BRCA1* and *BRCA2* P/LP variant carriers after their first breast cancer diagnosis [83].

All breast assessment risk tools have inherent limitations and biases. Moreover, it remains uncertain whether PRSs have enhanced the accuracy of these tools, as there is currently insufficient validation for the use of PRSs in clinical practice [3,88]. Each model is most appropriate for particular clinical situations and may have restricted relevance in specific patient populations [89]. Most risk assessment models and PRS studies used data from the European population and performed better than those developed on populations from other ancestry groups [73,90].

**Table 1:** Genetic risk models that include individuals with hereditary cancer.

Genetic Risk Model	Risk Factors Included	Exclusion Criteria	Strengths	Limitations
Tyrer–Cuzick/IBIS [76,84,91,92,93,94,95,96,97]	Age at menarche, age at first live birth, age at menopause, parity, height, BMI, atypical hyperplasia/lobular carcinoma in situ, use of HRT, benign breast disease, family history of breast and ovarian cancer in first- and second-degree relatives, and age at diagnoses, mammographic breast density, PRS_313_	None	The model combines genetic segregation model for familial risk and regression model for other risk factors; can be used in women younger than 35 years of age	Requires detailed genetic history and needs a computer program
Boadicea [77,84,85,98]	Hormonal, reproductive, and lifestyle risk factors, pedigree-level family history information, breast density, breast tumor pathology, and genetic testing results on rare P/LP variants in high- and moderate-risk genes (*BRCA1*, *BRCA2*, *PALB2*, *CHEK2*, and *ATM*), besides PRS_313_	None	Family history is not limited to certain relatives or degrees. Most suitable for high-risk women	Requires detailed family history
ASK2ME [87]	Age, gender, prior cancers, prior surgeries, and gene of interest	None	ASK2ME focuses on 28 cancer predisposition genes	The outputs do not currently include confidence intervals for the estimates and do not yet estimate the risk of a second cancer of a given organ when a prior cancer has occurred. ASK2ME assumes that for most genes, all P/LP variants in each gene will have the same implications for risk
BRCA-Crisk [83]	*BRCA1/BRCA2* carriers with breast cancer	Individuals diagnosed with stage IV breast cancer, or synchronous CBC, or who underwent CRRM	Access the absolute cumulative risk of developing CBC for *BRCA1* and *BRCA2* carriers with breast cancer	The analysis combined *BRCA1* and *BRCA2* breast cancer patients

BMI, body mass index; HRT, hormone replacement therapy; P/LP, pathogenic/likely pathogenic; PRS, polygenic risk score based on 313 variants; CBC, contralateral breast cancer; CRRM, contralateral risk-reducing mastectomy.

## 4. Primary and Secondary Prevention

Chemoprevention refers to the use of agents to reduce or delay the development of cancer [99]. Primary prevention represents an opportunity to intervene in modifiable factors before breast cancer develops. Primary chemoprevention is appropriate for both the general population and individuals at increased risk of developing the disease [100]. Depending on the stage at which they act, chemopreventives can be classified as primary or secondary. Primary chemopreventives target the prevention of tumor formation in at-risk populations. Secondary chemoprevention suppresses the transition of a tumor from a benign to a malignant phenotype (e.g., the use of agents by women with a preneoplastic lesion to lower the risk of developing an invasive carcinoma) [99]. For P/LP variant carriers, primary prevention aims to reduce breast cancer occurrence and includes lifestyle modifications, chemoprevention, and risk-reducing surgeries [8].

### 4.1. Lifestyle and Reproductive Factors

Overweight, obesity, a fatty diet, low physical activity, alcohol intake, and HRT have been associated with an increased risk of sporadic breast cancer [101]. Evidence regarding the impact of modifiable risk factors on breast cancer risk in women with hereditary cancer is scarce. Moreover, it remains unclear how lifestyle, hormonal, and reproductive factors affect breast cancer risk in women carrying moderate- and high-risk P/LP variants. For *BRCA1* and *BRCA2* carriers, few studies with small sample sizes, high heterogeneity, low quality, and varied measurements of exposure have assessed the impact of modifiable risk factors on breast cancer risk [102].

A systematic review examined the impact of dietary habits, weight changes, and physical activity on the risk of breast and ovarian cancer in women with P/LP variants in *BRCA1/BRCA2* P/LP [103]. The review found that maintaining a high-quality diet, losing at least 10 pounds during adulthood, and engaging in physical activity during adolescence and early adulthood may be associated with a reduced risk of breast cancer. Conversely, higher meat consumption and increased daily energy intake were linked to a greater risk of breast cancer. However, the authors highlighted a lack of robust evidence to support specific dietary or weight management recommendations tailored to women with BRCA1/BRCA2 P/LP variants. As a result, dietary and physical activity guidelines for these women should align with those recommended for the general population [103].

Previous evidence suggested that high serum levels of insulin-like growth factor I (IGF-I) were associated with increased *BRCA* penetrance for women with high genetic risk [104]. A multicenter prospective randomized controlled trial evaluated whether a Mediterranean dietary intervention with moderate restriction reduced IGF-I and other metabolic modulators of *BRCA* penetrance [105]. The intervention group significantly lowered IGF-I, total cholesterol, and triglycerides serum levels and reduced weight, waist circumference, and hip circumference compared with the control group [105]. Longer follow-up may assess the impact of a Mediterranean dietary intervention on breast cancer risk in *BRCA* P/LP variant carriers.

The ongoing randomized lifestyle intervention LIBRE trial is evaluating the impact of nongenetic modifiers on breast cancer risk associated with *BRCA* P/LP variant carriers [106]. The study aims to assess whether a structured intervention program, including endurance training paired with a Mediterranean diet, may improve both BMI and physical fitness. The long-term goals are to show a decrease in breast cancer risk, inhibited progression of the disease, and reduced cancer mortality rates in *BRCA* P/LP variant carriers following a healthy lifestyle [106].

Similarly to sporadic breast cancer, reproductive factors have been associated with the risk of hereditary breast cancer [107]. A case–control study included 1665 pairs of women with *BRCA1* (*n* = 1243 pairs) and *BRCA2* P/LP variants (*n* = 422 pairs) to assess the association between breastfeeding and breast cancer risk. In *BRCA1* P/LP variant carriers, the authors observed a reduction of 32% in breast cancer risk (OR 0.68; 95% CI 0.52–0.91; *p* = 0.008) for breastfeeding for at least one year and of 49% (OR 0.51; 95% CI 0.35–0.74) for over two years of breastfeeding [107].

Khincha et al. conducted the first study to evaluate the relationship between female reproductive factors and breast cancer risk in women with a pathogenic/likely pathogenic (P/LP) TP53 variant [107]. The study analyzed questionnaire data from 152 women participating in the National Cancer Institute’s Li–Fraumeni syndrome (LFS) study, including 85 women with breast cancer. The findings indicated that breastfeeding for a cumulative duration of at least one year was associated with a reduced risk of breast cancer (HR 0.49; 95% CI 0.26–0.89; *p* = 0.02). Conversely, women who had their first live birth after age 30 showed a modestly increased risk of breast cancer (HR 2.14; 95% CI 0.99–4.60; *p* = 0.05) [108].

As regards to HRT, the short-term use of HRT before the age of natural menopause does not seem to be associated with an increased risk beyond the baseline of breast cancer for women carrying *BRCA* P/LP variants [109]. A meta-analysis evaluated the association between the incidence of breast cancer and HRT in 1100 women carrying *BRCA1* and *BRCA2* P/LP variants and intact breasts who underwent bilateral salpingo-oophorectomy (RRSO) before the onset of natural menopause [109]. No significant association was observed between breast cancer risk and the use of HRT after RRSO (HR 0.98; 95% CI 0.63–1.52) [109].

### 4.2. Chemoprevention

Studies have shown that chemoprevention with tamoxifen, raloxifene, anastrozole, or exemestane may reduce breast cancer incidence in women at an increased risk of breast cancer [110]. In premenopausal women, tamoxifen is the only option for reducing breast cancer risk, whereas in postmenopausal women the options can be tamoxifen, raloxifene, anastrozole, or exemestane [111]. However, the utility of the chemoprevention for women under 35 years is unknown [112]. Evidence of tamoxifen’s benefit derives from trials conducted among predominantly postmenopausal women from the general population and studies of CBC in women carrying P/LP variants in *BRCA1* or *BRCA2* genes [110]. Consequently, data on the efficacy of chemopreventive agents for the primary prevention of breast cancer in women carrying P/LP variants are scarce [110].

The NSABP P-1 trial evaluated the efficacy of tamoxifen (versus placebo) in reducing breast cancer incidence in 13,388 women at increased risk of disease [112]. Subsequently, King et al. conducted a subgroup analysis to compare the occurrence of breast cancer among women with *BRCA1* or *BRCA2* P/LP variants who were received tamoxifen versus a placebo [113]. Among 288 women who developed breast cancer during the trial, 19 (6.6%) carried *BRCA1* (*n* = 8) or *BRCA2* P/LP variants (*n* = 11). In the BRCA1 P/LP variant group, five of the eight women who developed breast cancer were on tamoxifen, while three were in the placebo group (RR 1.67; 95% CI 0.32–10.7). Conversely, in the BRCA2 P/LP variant group, three of the eleven women who developed breast cancer were on tamoxifen, compared to eight in the placebo group (RR 0.38; 95% CI 0.06–1.56) [113]. However, the small sample size limited the ability to draw definitive conclusions.

A prospective cohort analysis evaluated the association between chemoprevention and the risk of breast cancer in unaffected women with *BRCA1* or *BRCA2* P/LP variants [114]. Of 4578 women, 137 reported tamoxifen use (3%), 83 reported raloxifene use (2%), and 12 used both agents (0.3%). After a mean follow-up of 6.8 years, there were 22 breast cancers among tamoxifen/raloxifene users and 71 cases among nonusers (10.9% of users vs. 14.3% of nonusers; HR 0.64; 95% CI 0.40–1.03; *p* = 0.07). The authors concluded by stating that further studies with a longer follow-up are necessary to establish the efficacy of chemoprevention in *BRCA1* or *BRCA2* P/LP variant carriers [114]. Although the efficacy of tamoxifen for the prevention of primary breast cancer in *BRCA* P/LP variant carriers is uncertain, tamoxifen use has been correlated with a reduced incidence of CBC [115].

Studies with low-dose tamoxifen have shown lower toxicity and similar efficacy than higher doses of tamoxifen [116,117]. However, data assessing the efficacy of low-dose tamoxifen for the chemoprevention of hereditary breast cancer are lacking. Higher concentrations of sex hormone-binding globulin (SHBG) have been associated with a reduced risk of breast cancer [118]. The TOLERANT (Low Dose TamOxifen and LifestylE Changes for bReast cANcer prevenTion, NCT06033092) trial aims to verify whether low-dose tamoxifen increases circulating levels of SHBG more than lifestyle intervention with or without intermittent caloric restriction after six months in women at an increased risk of breast cancer. The study aims to include women carrying a germline P/LP variant (*BRCA1*, *BRCA2*, or *PALB2*), or >5% Tyrer–Cuzick breast cancer risk at ten years, or with a previous diagnosis with intraepithelial neoplasia within the last three years [119].

The French Liber Trial compared the efficacy of letrozole compared to a placebo for primary prevention of breast cancer in postmenopausal women carrying *BRCA1* or *BRCA2* P/LP variants [120]. After a median follow-up of 72 months, the 5-year invasive breast cancer-free survival rates were comparable to the overall population: 92% for the placebo and 91% for letrozole (HR 0.83; 95% CI 0.3–2.3). However, the study was underpowered (170 included out of 270 expected), and the dropout rate was high (around 40% in both arms). Another limitation of the study was the inclusion of only postmenopausal women, limiting the applicability to younger carriers [120].

Aromatase inhibitor use has also shown a decrease in the risk of CBC [121]. A retrospective study included 935 subjects (53 *BRCA1* and 94 *BRCA2* P/LP variant carriers), of whom 72% (*n* = 676) received tamoxifen and 43% (*n* = 405) were given aromatase inhibitors. There were 66 cases of CBC, of which 10% (15/147) occurred in *BRCA* P/LP variant carriers and 6,5% (51/788) in noncarriers. The results showed that aromatase inhibitor use was associated with a decreased risk of CBC (HR 0.44; *p* = 0.004) regardless of the *BRCA* status. However, tamoxifen use was not associated with a reduced risk of CBC in this analysis [121].

For *CDH1* P/LP variant carriers, the IGCLC (International Gastric Cancer Linkage Consortium) guidelines recommend chemoprevention with selective estrogen receptor modulators or aromatase inhibitors to prevent the occurrence of lobular carcinoma [122].

Although specific studies on chemoprevention for lobular breast carcinoma are not available, evidence from LCIS studies suggests a greater benefit of chemoprevention in reducing the risk of invasive lobular carcinoma [123].

The uptake of preventive strategies among women carrying *BRCA1* and *BRCA2* P/LP variants is relatively low [124]. Metcalfe et al. studied the use of risk-reducing bilateral mastectomy (RRM), RRSO, tamoxifen, and screening via magnetic resonance imaging (MRI) and mammography in a cohort of 2677 women with these variants. Approximately 50% relied solely on screening, while 57% underwent RRSO. Among the 1383 women without a prior breast cancer diagnosis, only 18% underwent RRM. Of those who did not undergo RRM, tamoxifen use was reported by only 5.5% and raloxifene by 2.9%. The adoption of preventive measures also varied significantly across countries. Women from the United States were most likely to use tamoxifen or raloxifene, while none of the participants from Norway, Italy, Holland, or France reported using either drug. Moreover, in women without breast cancer, tamoxifen use was higher among women who underwent RRSO than those who did not [124].

In breast cancer, two poly(ADP-ribose) polymerase (PARP) inhibitors, olaparib (OlympiAD trial) and talazoparib (EMBRACA trial), have been approved to treat germline *BRCA* carriers with metastatic HER2-negative breast cancer [125,126]. Olaparib has also been approved for adjuvant treatment of germline *BRCA* carriers’ HER2-negative breast cancer with an elevated risk of recurrence (OlympiA trial) [127]. PARP inhibitors have also been evaluated in the neoadjuvant setting [128,129]. However, the role of PARP inhibitors in breast cancer prevention is still unknown. About 46% of patients in the Olympia trial underwent bilateral mastectomies, making the evaluation of CBC incidence more difficult. Interestingly, after a median follow-up of 3.5 years, the frequency of regional and local ipsilateral recurrences was slightly lower in the olaparib arm (0.7% vs. 1.5% and 0.8% vs. 1.2%, respectively). The same occurred for invasive CBC and second primary malignancies (0.9% vs. 1.3% and 1.2% vs. 2.3%, respectively) [127]. Longer follow-up of the Olympia trial may report the incidence of second breast cancer and other cancers and the long-term safety of olaparib [130]. However, the adverse event profile, such as hematological toxicity, and the need for regular monitoring may limit the use of PARP inhibitors as preventive agents [131].

A promising agent specifically for *BRCA1* carriers is denosumab, an anti-receptor activator of nuclear factor-kB-ligand (RANKL) monoclonal antibody approved for treating osteoporosis and preventing skeletal-related events caused by bone metastasis [132]. RANKL binds to RANK on mammary epithelial cells and stimulates their proliferation, contributing to mammary tumorigenesis [132]. The RANK/RANKL system is dysregulated in *BRCA1* P/LP variant carriers [132]. Kotsopoulos et al. reported that exposure to progesterone (but not estrogen) was a risk factor for *BRCA1*-associated breast cancer after oophorectomy [133]. Denosumab targets the progesterone pathway by blocking the RANKL/RANK signaling pathway [134]. In mouse models, denosumab delayed onset, reduced incidence, and attenuated the progression of *BRCA1* mutation-driven breast cancer [134]. Table 2 presents the ongoing trials assessing different prevention strategies in hereditary breast cancer.

### 4.3. Risk-Reducing Surgeries

Women carrying P/LP variants associated with hereditary cancer syndromes may have an extremely high risk of breast cancer and other cancers and may benefit from MRRM and other risk-reducing surgeries, especially [8,137].

#### 4.3.1. Risk-Reducing Bilateral Salpingo-Oophorectomy and Breast Cancer Risk Reduction

There is no evidence showing that screening for ovarian and tubal cancers may reduce disease-specific deaths, including for *BRCA1/2* P/LP variant carriers [138]. The only strategy shown to decrease the incidence and mortality from ovarian and tubal cancer in *BRCA1/BRCA2* P/LP variant carriers was RRSO [139]. For breast cancer primary prevention, the rationale of RRSO is to reduce exposure to ovarian hormones, decreasing breast cancer occurrence [140].

A systematic review found no association between RRSO and the risk of primary breast cancer (RR 0.84; 95% CI 0.59–1.21) or CBC (RR 0.95; 95% CI 0.65–1.39), even when considering combined *BRCA1/BRCA2* P/LP or only *BRCA1* P/LP variant carriers (RR 0.89; 95% CI 0.68–1.17 and RR 0.85; 95% CI 0.59–1.24, respectively) [141]. However, a subgroup analysis showed an association between RRSO and risk-reduction in primary breast cancer in *BRCA2* P/LP variant carriers (RR 0.63; 95% CI 0.41–0.97) but not for CBC (RR 0.35; 95% CI 0.07–1.74). In addition, the analyses reported reduced breast cancer-specific mortality in breast cancer-affected women carrying *BRCA1/BRCA2* P/LP variants combined (RR 0.26; 95% CI 0.18–0.39) [141].

An international longitudinal cohort study evaluated the association between prophylactic bilateral oophorectomy (with and without salpingectomy) and all-cause mortality among women carrying P/LP variants in *BRCA1* and *BRCA2* [142]. The study enrolled 4332 women, of whom 2932 underwent a preventive oophorectomy. After a mean follow-up of nine years, the age-adjusted HR for all-cause mortality associated with oophorectomy was 0.32 (95% CI 0.24–0.42; *p* < 0.001). For *BRCA1* P/LP variant carriers, the age-adjusted HR was 0.29 (95% CI 0.20–0.38; *p* < 0.001), whereas for *BRCA2* P/LP variant carriers, it was 0.43 (95% CI 0.22–0.90; *p* = 0.03). Among *BRCA1* P/LP variant carriers, the cumulative mortality from all causes to age 75 years was lower for women who underwent prophylactic oophorectomy at age 35 compared to those who did not (25% vs. 62%, respectively). The results estimated the all-cause cumulative mortality for *BRCA2* P/LP variant carriers to be 14% for women who underwent a preventive oophorectomy and 28% for those who did not. Furthermore, breast cancer accounted for 1.02% (30 out of 2932) of deaths in women who underwent oophorectomy and for 2.00% (28 out of 1400) in those who did not [142]. Despite the suggestive association between prophylactic bilateral oophorectomy and reduced all-cause mortality, the study’s observational design precludes definitive conclusions.

#### 4.3.2. Risk-Reducing Mastectomy in Unaffected Individuals

Women carrying P/LP variants should be aware of their potential and long-term risks of developing breast cancer and the limitations of the current evidence when considering RRM [143]. Gene categorization by phenotype penetrance varies among guidelines, resulting in different management recommendations [144]. Most data regarding RRM decision-making come from studies that included *BRCA1* and *BRCA2* healthy carriers [145]. RRM has decreased the relative risk of breast cancer in healthy *BRCA1* and *BRCA2* P/LP variant carriers by 90–95% [145].

However, the impact on survival is still uncertain [146]. A prospective study conducted by Metcalfe et al. included 1654 women carrying P/LP variants in *BRCA1* or *BRCA2* to assess the impact of RRM on breast cancer mortality [146]. The study matched one woman who underwent RRM (*n* = 827) to one who did not (*n* = 827) on birth year, gene, and country. After a follow-up of 6.3 years, the occurrence of incident breast cancer was lower in the RRM arm compared to the control arm (2.4% vs. 12.1%, respectively; HR = 0.20; 95% CI 0.12–0.32; *p* < 0.0001). Two women in the RRM arm and seven in the control arm died from breast cancer (HR 0.26; 95% CI 0.05–1.35; *p* = 0.11). Moreover, the probability of dying from breast cancer within 15 years after RRM was low (0.95%) [146]. However, further follow-up is necessary to estimate the mortality reduction accurately.

Most recommendations for *PALB2* P/LP variant healthy carriers consider RRM as an option and suggest personalized risk assessment to guide decisions [147]. For *TP53* healthy female carriers, the guidelines recommend discussing RRM [8,148]. The IGCLC guidelines recommend considering RRM for women carrying *CDH1* P/LP variants aged between 30 and 60 [122]. For *CDH1* P/LP variant carriers, data regarding the risk of CBC are scarce [149].

The benefits of RRM in carriers of moderate- or low-penetrance P/LP variants without a strong family history remain unclear [150]. For individuals with a *PTEN* P/LP variant, the guidelines recommend discussing the option of RRM based on family history [8].

#### 4.3.3. Contralateral Risk-Reducing Mastectomy

Evidence regarding surgical management and surveillance strategies among patients with breast cancer carrying a P/LP variant is limited. Young women with breast cancer have a significant risk of CBC and ipsilateral breast cancer, warranting discussion of prophylactic mastectomy and contralateral RRM (CRRM) [151]. When discussing CRRM, it is essential to provide P/LP variant carriers with the absolute risk estimates of CBC and balance the pros and cons of prophylactic surgery [151,152].

For *BRCA1* and *BRCA2* P/LP variant carriers, the evidence shows a strong association with the risk of CBC [8]. The 20-year cumulative risk estimates of CBC are around 30–40% for *BRCA1* and 25% for *BRCA2* P/LP variant carriers. Moreover, the 15-year cumulative risk for premenopausal *BRCA1* and *BRCA2* P/LP variant carriers may exceed 20% [23,47].

The risk assessment of CBC and the benefit of CRRM in *BRCA1* and *BRCA2* P/LP variant carriers should consider age at diagnosis, family history of breast cancer, the overall prognosis from the first breast cancer or other cancers, the ability of the patient to undergo proper breast surveillance, comorbidities, and life expectancy [151]. The younger the age at first breast cancer diagnosis, the higher the absolute risk of CBC [23]. In addition, for *BRCA1* and *BRCA2* P/LP variant carriers, the BRCA-Crisk model may help to discriminate women with a high risk from those with a low risk of CBC, helping them to decide between unilateral versus bilateral mastectomy [83].

Yadav et al. reported CBC risk estimates among breast cancer survivors carrying P/LP variants in the *ATM*, *BRCA1*, *BRCA2*, *CHEK2*, and *PALB2* genes from the CARRIERS study [47]. The results showed an increased risk of CBC for *BRCA1*, *BRCA2*, and *CHEK2* P/LP variant carriers (HR 1.9). The 10-year cumulative incidence estimated for *PALB2* P/LP carriers with primary ER-negative breast cancers was high, around 33%. Conversely, *ATM* P/LP variant carriers did not present an elevated risk of CBC. The 10-year cumulative incidence of CBC estimates was higher for premenopausal than postmenopausal women carrying a P/LP variant in *BRCA1* (33% vs. 12%), *BRCA2* (27% vs. 9%), and *CHEK2* (13% vs. 4%, respectively). In addition, *BRCA2* was the only gene associated with an increased risk of CBC in postmenopausal women (HR 3.0: HR 1.7–5.2; *p* < 0.001). Interestingly, for *PALB2*, the risk of CBC was increased if the primary breast cancer was ER-negative (HR 2.9; 95% CI 1.4–6.4; *p* = 0.006), whereas it was increased for *CHEK2* if the primary breast cancer was ER-positive (HR 2.0; 95% CI 1.1–3.5; *p* = 0.02) [47].

Morra et al. assessed the association of protein-truncating variants and rare missense variants in nine genes (*ATM*, *BARD1*, *BRCA1*, *BRCA2*, *CHEK2*, *PALB2*, *RAD51C*, *RAD51D*, and *TP53*) with the risk of CBC [153]. The analysis included 34,401 women of European ancestry diagnosed with breast cancer, and the median follow-up was 10.9 years. The results showed an increased risk of CBC in women carrying protein-truncating variants and rare missense variants in *BRCA1* (HR 2.88), *BRCA2* (HR 2.31), and *TP53* (HR 8.29), and protein-truncating variants in *CHEK2* (HR 2.25) and *PALB2* (HR 2.67) [153].

As the risk of CBC may vary according to different P/LP variants, the impact of CRRM on decreasing the risk of CBC depends on the risk of individual genes [8]. There is limited evidence regarding the risk of CBC in women carrying P/LP variants in moderate-penetrance genes, and the decision to undergo CRRM should be individualized and consider age at diagnosis and family history [65,151].

A cohort study assessed the benefit of CRRM in affected women carrying a P/LP variant in *BRCA1* [154]. The study included 2482 patients with invasive breast cancer stage I-III. The mean follow-up was 8.9 years. Approximately 11% of the patients developed CBC: 0.8% of the patients who underwent CRRM, 10.8% of those who had breast-conserving surgery (BCS), and 11.4% of those who underwent unilateral mastectomy. The results showed that *BRCA1* carriers with breast cancer who underwent CRRM were less likely to develop CBC (*p* < 0.0001), and women who developed CBC were twice as likely to die of breast cancer (HR 2.22; 95% CI 1.49–3.32; *p* < 0.0001). In addition, CRRM was not significantly associated with a reduction in mortality compared to BCS (HR 0.83; *p* = 0.52). The mortality rate was higher in patients who underwent unilateral mastectomy (15.2%) than in those who underwent CRRM (7.4%) or BCS (6.9%; *p* < 0.0001). The 15-year breast cancer-specific survival rates were 88,7% for CRRM, 86,2% for BCS, and 78,7% for unilateral mastectomy. However, there were significant differences across the three groups. Patients in the CRRM group tended to be younger (*p* < 0.0001), were more likely to undergo oophorectomy (*p* < 0.0001), and were less likely to receive radiotherapy (*p* < 0.0002), chemotherapy (*p* < 0.0002), and tamoxifen (*p* < 0.0002). In contrast, patients who underwent unilateral mastectomy had larger tumors (*p* < 0.0001) and more node-positive disease (*p* = 0.03) [154].

The discussion about CRRM should consider the individual absolute risk of CBC. The ASCO/ASTRO/SSO guidelines recommend offering CRRM for women with breast cancer carrying a *BRCA1* or *BRCA2* P/LP variant and who have been treated or are being treated with unilateral mastectomy [151]. The recommendation is based on the reduced risk of CBC associated with CRRM; however, evidence showing the survival benefit is still insufficient [151]. Table 3 presents the NCCN, ASCO/ASTRO/SSO, NICE, ESMO, St. Gallen, and IGCLC recommendations for RRM/CRRM in moderate- and high-penetrance P/LP variant carriers. Table 4 compares the recommendations of different guidelines for risk-reducing mastectomy in unaffected individuals (RRM) and contralateral risk-reducing mastectomy in affected individuals (CRRM).

### 4.4. Surveillance Programs

Secondary prevention aims to detect breast cancer early through intensified surveillance with breast imaging to enhance the chances of cure [157]. Several clinical practice guidelines have developed recommendations for hereditary breast cancer screening [158]. Although some recommendations may differ in detail, most are consistent and focus on annual mammography and MRI [158].

Most guidelines on breast cancer surveillance for *BRCA1* and *BRCA2* P/LP variant carriers recommend starting breast screening at 25 years or individualizing the screening according to family history if a breast cancer diagnosis before age 30 is present [8,158]. Annual screening with MRI should start between 25 and 29 years, with annual mammography and MRI combined between 30 and 75 years and individualized management after 75 years [8]. For *PALB2* P/LP variant carriers, the surveillance for breast cancer should be equivalent to that for *BRCA1* and *BRCA2* P/LP variant carriers [147].

A prospective study evaluated the association between annual MRI surveillance and the risk of breast cancer mortality among women carrying *BRCA1* or *BRCA2* P/LP variants [159]. The cohort study included 1442 women carrying a P/LP variant in *BRCA1* and 314 in *BRCA2*. After a mean follow-up of 9.2 years, the HR for breast cancer mortality associated with MRI surveillance was 0.23 (95% CI 0.11–0.48; *p* = 0.001). For *BRCA1*, the HR was 0.20 (95% CI 0.01–0.43; *p* < 0.001), whereas for *BRCA2* P/LP variant carriers, it was 0.87 (95% CI 0.10–17.25; *p* = 0.93). At 20 years, the risk of breast cancer mortality was lower for women who underwent MRI surveillance compared to those who did not (3.2% vs. 14.9%) [159].

Another prospective cohort study assessed the efficacy of intensified surveillance with bi-annual dynamic contrasted-enhanced MRI in women with a cumulative lifetime breast cancer risk ≥20% and/or tested positive for a P/LP variant in a known breast cancer susceptibility gene [160]. The screening performed well, especially in women at high risk of aggressive *BRCA1*-associated breast cancer, by detecting invasive tumors ≤1 cm without nodal involvement and effectively avoiding interval invasive cancers with low recall rates. In addition, annual mammography did not add a screening benefit when performed with bi-annual MRI screening [160].

The most effective management for women with moderate-penetrance P/LP variants needs to be defined [29]. An analysis used breast cancer microsimulation models from the Cancer Intervention and Surveillance Modeling Network to compare different screening strategies for *ATM*, *CHEK2*, or *PALB2* P/LP variant carriers [161]. The analysis estimated age-specific breast cancer risks using aggregated data from the CARRIERS consortium in 12 population-based studies. The results suggested that annual MRI screening from 30 to 35 years, followed by annual mammography at 40 years, might reduce breast cancer mortality by over 50% for women with *ATM, CHEK2*, and *PALB2* P/LP variants [161].

A study that assessed the effectiveness of surveillance in 38 women with *PTEN* hamartoma tumor syndrome showed that annual breast cancer surveillance with MRI starting at age 25 enabled the detection of early-stage breast cancer. The surveillance-detected breast cancers were all T1 and N0, whereas outside surveillance-detected breast cancers were more often tumors staged higher than T2 (60%) and lymph node-positive disease (45%) [162].

For LFS, there is no consensus on the use of mammography for breast cancer screening [163]. Considering the early-onset breast cancer risk in *TP53* P/LP variant carriers, most guidelines recommend an annual breast MRI with and without contrast from the ages of 20–29, annual MRI and mammography screening between the ages of 30 and 75, and individualized screening after 75 years [6,40,148]. The inclusion of mammography took into account that MRI cannot detect microcalcification optimally [163]. Other guidelines recommend an annual MRI, considering annual mammography ± ultrasound if MRI is unavailable [148,164,165]. There is no evidence evaluating the potential detrimental effect of ionizing radiation from mammography used for screening in *TP53* P/LP variant carriers [166]. However, there is a concern about the long-term risk and cumulative ionizing radiation exposure in young women [166]. Consequently, other guidelines do not recommend mammography for *TP53* P/LP carriers [167]. The European Reference Network Genturis guidelines recommend an annual MRI from 20 to 65 years without mammography [168]. For those families in which there was already a case of breast cancer at or around 20, awareness and screening should begin five to ten years before the earliest age of onset [148]. In addition, for affected individuals treated for breast cancer who have not had an RRM, screening should include an annual MRI and mammography [8].

The IGCLC guidelines for women carrying a P/LP variant in *CDH1* annual breast MRI with contrast from the age of 30, recommend annual mammography from the ages of 35–40 and annual ultrasound for women unable to have an MRI or without access to MRI [122].

Screening with ultrasound for women with an increased risk of breast cancer in combination with MRI or mammography has not shown added value [169]. However, ultrasound screening may be considered a supplemental tool for women carrying *BRCA* P/LP variants with contraindications to MRI screening [169].

Contrast-enhanced mammography (CEM) has emerged as a viable alternative to contrast-enhanced breast MRI, and it may increase access to vascular imaging while reducing examination costs [170]. Digital breast tomosynthesis combined with digital mammography (two-dimensional, 2D) may improve cancer detection and reduce false-positive callback rates [171]. However, most guidelines do not recommend tomosynthesis for breast cancer screening in P/LP variant carriers.

There is no evidence to support continued breast imaging after RRM [21]. For P/LP variant carriers who underwent RRM, screening should include a basal MRI in the first year after RRM to evaluate residual breast tissue, followed by an annual clinical examination [21]. Further decisions on imaging screening should be individualized [21]. Table 5 presents the NCCN, NICE, ESMO, and IGCLC recommendations for breast cancer screening for moderate- and high-penetrance P/LP variant carriers.

## 5. Conclusions and Future Perspectives

In the following years, we expect to identify an increasing number of individuals carrying P/LP variants associated with hereditary breast cancer because of the adoption of broader criteria for genetic testing and the growing use of expanded genetic panels. Both genetic and non-genetic factors influence the penetrance of hereditary breast cancer. Not all individuals with P/LP variants will develop the disease. Various factors—such as age, family history, lifestyle, specific germline variants, polygenic risk scores, and reproductive and hormonal influences, as well as other genetic and yet unknown factors—contribute to the overall risk. Current models of risk assessment often oversimplify these complexities, leading to inconsistencies in the adoption of personalized risk-reduction strategies. Despite existing consensus guidelines for prevention, these frameworks are often limited by a one-size-fits-all approach that inadequately accounts for individual variability. A major challenge lies in the gaps in understanding how specific genomic variations translate into phenotypic differences. Therefore, even in hereditary breast cancer, we should individualize risk assessment to adopt personalized risk-reduction strategies. There is a pressing need to integrate genotypic and phenotypic data with lifestyle and clinical factors more effectively, aiming for a more balanced approach to risk–benefit considerations and quality of life. This includes improving the integration of genotypic/phenotypic analysis, lifestyle, and clinical factors to better balance risk–benefit considerations and quality of life in the implementation of surveillance and preventive interventions. Advances in genomic profiling and computational tools may offer deeper insights into these complex interactions, ultimately guiding more precise and effective strategies for managing hereditary breast cancer risk. This revision incorporates a more critical and analytical tone, emphasizing current limitations, challenges, and areas for improvement while highlighting the potential for future advancements. Further prospective studies are necessary to assess the impact of lifestyle modifications and chemoprevention in hereditary breast cancer.

## Figures and Tables

**Table 2:** Ongoing studies assessing preventive strategies in hereditary breast cancer.

ID and Reference	Study	Inclusion Criteria	Primary Objective	Intervention
NCT04711109 [135]	BRCA-P: A Randomized, Double-Blind, Placebo-Controlled, Multi-Center, International Phase 3 Study to Determine the Preventive Effect of Denosumab on Breast Cancer in Women Carrying a *BRCA1* Germline Mutation	Women with a confirmed deleterious or likely deleterious *BRCA1* germline mutation (variant class 4 or 5)	To evaluate the reduction in the risk of any breast cancer (invasive or ductal carcinoma in situ [DCIS]) in women with germline *BRCA1* mutation who are treated with denosumab compared to placebo	Denosumab vs. placebo
NCT06033092 [119]	Low Dose TamOxifen and LifestylE Changes for bReast cANcer prevenTion: a Randomized Phase II Biomarker Trial in Subjects at Increased Risk	Women at increased risk of breast cancer (i.e., healthy participants carriers of a germline pathogenic/likely pathogenetic variant in at least one of the following genes: *BRCA1*, *BRCA2*, *PALB2*, *ATM*, *CHEK2*, *CDH1*, *RAD51C or RAD51D*, or with >5% breast cancer risk at 10 years, using the Tyrer–Cuzick scale	To verify whether low-dose tamoxifen (LDT) increases circulating levels of SHBG more than lifestyle intervention (LI) with or without intermittent caloric restriction (ICR) after 6 months in women at increased risk of breast cancer in Cancer Surveillance Consortium risk models or with a recently resected intraepithelial neoplasia of the breast (IEN)	Arm 1: Low-dose tamoxifen (LDT), i.e., 10 mg every other day; Arm 2: Low-dose tamoxifen (LDT) + intermittent caloric restriction (ICR); Arm 3: Lifestyle intervention (LI) using a step counter; Arm 4: Lifestyle intervention (LI) using a step counter + intermittent caloric restriction (ICR)
LIBRE-2-20150626 [136]	Prospective Randomized Multicenter Trial to Assess the Efficacy of a Structured Physical Exercise Training and Mediterranean Diet in Women with *BRCA1/2* Mutations	Proven pathogenic *BRCA1/2* mutation	Mediterranean Diet Adherence Screener (MEDAS) Score (Questionnaire); body mass index (BMI); ventilatory threshold 1 (VT1) in spiroergometry	Structured exercise training plus mediterranean diet vs. control

**Table 3:** Risk-reducing surgeries for moderate- and high-penetrance P/LP variant carriers.

Gene	Guidelines						
	NCCN [8]	ASCO/ASTRO/SSO * [151]	NICE [155]	ESMO [21]	St. Gallen ** (2021) [22]	St. Gallen ** (2023) [156]	IGCLC [122]
*BRCA1*	Discuss option of RRM.	For women *BRCA1* or *BRCA2* carriers with breast cancer who have been treated with unilateral mastectomy, CRRM should be offered.	Consider RRM for women at high risk.	Consider RRM as an option.	Most of the panel suggested RRM for *BRCA1*, *BRCA2*, *PALB2*, and *TP53* carriers aged ~40 years. For women aged ~60 years, the panel was balanced, 46% suggested RRM, and 54% surveillance.	Most of the panel recommended RRM for *BRCA1* carriers and for premenopausal *BRCA2* carriers. For *BRCA2* postmenopausal carriers, the panel was balanced (42% favored RRM and 38% surveillance).	–
*BRCA2*							–
*PALB2*	Consider *PALB2* as a high-penetrance gene. Discuss the option of RRM.	Consider *PALB2* as a moderate-penetrance gene. No evidence available regarding the role of CRRM.	_	Consider *PALB2* as a high penetrance gene. Consider RRM as an option.		For premenopausal carriers, the panel was balanced (42% favored RRM and 38% surveillance). Most of the panel recommended surveillance for postmenopausal carriers.	–
*TP53*	Discuss option of RRM.	For women *TP53* carriers with breast cancer who have been treated with unilateral mastectomy, CRRM should be offered.	_	Consider RRM.		–	–
*CDH1*	Consider *CDH1* as a high-penetrance gene. Discuss the option of RRM.	For women *CHD1* carriers with breast cancer who have been treated with unilateral mastectomy, CRRM should be offered.	_	Consider RRM.	Most of the panel recommended surveillance over RRM.	–	Consider RRM, not under 30 years nor generally after 60 years.
*CHEK2*	Evidence insufficient for RRM; manage based on family history.	Consider *CHEK2* as a moderate-penetrance gene. No evidence available regarding the role of CRRM, aside from some data on *CHEK2 1100delC.*	_	Evidence insufficient for RRM.		Most of the panel recommended surveillance over RRM.	_
*STK11*	Consider *STK11* as a high penetrance gene. Discuss option of RRM.	–	_	Consider RRM.		Most of the panel recommended surveillance over RRM.	–
*BARD1*	Evidence insufficient for RRM; manage based on family history.	_	_	_		Most of the panel recommended surveillance over RRM.	_
*ATM*	Evidence insufficient for RRM; manage based on family history.	Consider *ATM* as a moderate-penetrance gene. No evidence available regarding the role of CRRM.	_	Evidence insufficient for RRM.	The entire panel recommended surveillance.	Most of the panel recommended surveillance over RRM.	–
*NF1*	Evidence insufficient for RRM, manage based on family history.	–	_	–		–	–
*PTEN*	Consider *PTEN* as a high-penetrance gene. Discuss option of RRM in individuals with P/LP variants identified. For those with clinical CS/PHTS syndrome, consideration of risk-reducing surgery should be based on family history.	–	_	Consider RRM.	–	–	–

NCCN, National Comprehensive Cancer Network; NICE, National Institute for Health and Care Excellence; ESMO, European Society for Medical Oncology; ASCO, American Society of Clinical Oncology; ASTRO, American Society for Radiation Oncology; SSO, Society of Surgical Oncology; IGCLC, International Gastric Cancer Linkage Consortium; RRM, risk-reducing bilateral mastectomy; CRRM, contralateral risk-reducing mastectomy; P/LP, pathogenic/likely pathogenic; CS/PHTS, Cowden syndrome; PTEN, hamartoma tumor syndrome. * ASCO/ASTRO/SSO guidelines consider only CRRM for affected carriers. ** The experts from the St. Gallen conference did not distinguish unaffected from affected carriers nor prophylactic RRM from therapeutic mastectomy with CRRM; –: Recommendations not provided.

**Table 4:** Guidelines’ recommendations for risk-reducing mastectomy in unaffected individuals (RRM) and contralateral risk-reducing mastectomy in affected individuals (CRRM).

Genes	Guidelines											
	NCCN [8]		ESMO [21]		NICE [155]		St. Gallen 2023 [22]		ASCO/ASTRO/SSO [151]		IGCLC [122]	
	RRM	CRRM	RRM	CRRM	RRM	CRRM	RRM	CRRM	RRM	CRRM	RRM	CRRM
*BRCA1*	**✔**	–	**✔**	–	**✔**	–	**✔**	–	–	**✔**	–	–
*BRCA2*	**✔**	–	**✔**	–	**✔**	–	**✔**	–	–	**✔**	–	–
*PALB2*	**✔**	–	**✔**	–	–	–	**✔**	–	–	**?**	–	–
*TP53*	**✔**	–	**✔**	–	–	–	–	–	–	**✔**	–	–
*CDH1*	**✔**	–	**✔**	–	–	–	❌	–	–	**✔**	**✔**	–
*CHEK2*	**?**	–	**?**	–	–	–	❌	–	–	**?**	–	–
*STK11*	**✔**	–	**✔**	–	–	–	–	–	–	–	–	–
*ATM*	**?**	–	**?**	–	–	–	❌	–	–	–	–	–
*BARD1*	**?**	–	**?**	–	–	–	–	–	–	–	–	–
*NF1*	**?**	–	**?**	–	–	–	–	–	–	–	–	–
*PTEN*	**✔**	–	**✔**	–	–	–	–	–	–	–	–	–

NCCN, National Comprehensive Cancer Network; NICE, National Institute for Health and Care Excellence; ESMO, European Society for Medical Oncology; ASCO, American Society of Clinical Oncology; ASTRO, American Society for Radiation Oncology; SSO, Society of Surgical Oncology; IGCLC, International Gastric Cancer Linkage Consortium; RRM, risk-reducing bilateral mastectomy; CRRM, contralateral risk-reducing mastectomy. **✔**: Guidelines recommend RRM or CCRM. **?**: Recommendations according to family history. ❌: Guidelines do not recommend RRM or CRRM. –: Recommendations not provided.

**Table 5:** Breast cancer screening recommendations for moderate- and high-penetrance P/LP variant carriers.

Gene	Guidelines			
	NCCN [8]	NICE [155]	ESMO [21]	IGCLC [122]
*BRCA1*	Age 25–29 years, annual breast MRI screening with and without contrast (or mammography, only if MRI is unavailable) or individualized based on family history if a breast cancer diagnosis before age 30 is present. Age 30–75 years, annual mammography and breast MRI screening with and without contrast. Age >75 years, management should be considered on an individual basis. For individuals with a *BRCA* P/LP variant who are treated for breast cancer and have not had a bilateral mastectomy, screening with annual mammography and breast MRI. For male breast cancer, consider annual mammography, especially for those with *BRCA2* P/LP variants in whom the lifetime risk of breast cancer is up to 7%, starting at age 50 or 10 years before the earliest known male breast cancer in the family.	Consider annual mammography in women aged 30–39 years Consider annual breast MRI for women aged 30–49 years	Intensified screening should start at age 30, or 5 years younger than the youngest family member with breast cancer. Imaging should be carried out at 6-monthly intervals. If MRI is not available for 6-monthly screening, consider the following: - In carriers 30–39 years of age, ultrasound with or without mammography. - In carriers ≥40 years of age, mammography with or without ultrasound.	–
*BRCA2*			Intensified screening should start at age 30, or 5 years younger than the youngest family member with breast cancer. Imaging should be performed annually.	–
*PALB2*	Annual mammography and breast MRI with and without contrast at 30 years.	–	Intensified screening should start at age 30, or 5 years younger than the youngest family member with breast cancer. Annual breast MRI from age 20–29 years. Annual breast MRI and/or mammography at age 30–75 years.	_
*TP53*	Age 20–29 years, annual breast MRI screening with and without contrast. Age 30–75 years, annual breast MRI screening with and without contrast and mammography. Age >75 years, management should be considered on an individual basis. For individuals with a *TP53* P/LP variant who are treated for breast cancer, and who have not had a bilateral mastectomy, screening with annual breast MRI and mammography should continue as described above.	–	Annual breast MRI at age 20–75 years. If MRI is not available, consider mammography.	_
*CDH1*	Annual mammography and consider breast MRI with and without contrast starting at age 30 years.	–	Annual breast MRI from age 20–29 years. Annual breast MRI and/or mammography at age 30–75 years.	Annual MRI from age 30 years. Annual mammography from age 35–40.
*CHEK2*	Annual mammography at age 40 years and consider breast MRI with and without contrast starting at age 30–35 years.	_	Annual breast MRI from age 20–29 years. Annual breast MRI and/or mammography at age 30–75 years.	_
*ATM*	Annual mammography at age 40 years and consider breast MRI with and without contrast starting at age 30–35 years.	–	Annual breast MRI (no evidence regarding the age of onset).	_
*PTEN*	Annual mammography and breast MRI screening with and without contrast starting at age 30 years or 10 years before the earliest known breast cancer in the family (whichever comes first). Age >75 years, management should be considered on an individual basis. For individuals with a *PTEN* P/LP variant who are treated for breast cancer, and have not had a bilateral mastectomy, screening with annual mammography and breast MRI.	–	Annual breast MRI and/or mammography at age 30–75 years.	_
*STK11*	Annual mammography and breast MRI with and without contrast starting at age 30 years.	–	Annual breast MRI from age 20–29 years. Annual breast MRI and/or mammography at age 30–75 years.	_
*BARD1*	Annual mammography and consider breast MRI with and without contrast starting at age 40 years.	–	–	_
*NF1*	Annual mammography starting at age 30 years and consider breast MRI with and without contrast from ages 30–50 years.	–	_	_

NCCN, National Comprehensive Cancer Network; NICE, National Institute for Health and Care Excellence; ESMO, European Society of Medical Oncology; IGCLC, International Gastric Cancer Linkage Consortium; MRI, magnetic resonance imaging; –: Recommendations not provided.
